# Temporal evolution of carbon footprint and emission reduction pathways of major crops in Inner mongolia under China’s dual-carbon goals

**DOI:** 10.1371/journal.pone.0353558

**Published:** 2026-07-16

**Authors:** Jiuwei Chi, Na Liu, Fang Wang, Jingran Yu, Xiao Zhang, Chuang Yue, Rifu Bada

**Affiliations:** 1 Department of Chemistry and Environmental Engineering, Hetao College, Bayannur, Inner Mongolia, China; 2 College of Agronomy, Inner Mongolia Agricultural University, Hohhot, Inner Mongolia, China; MRC Unit The Gambia at LSHTM, GAMBIA

## Abstract

As an important grain-producing region in northern China, the Inner Mongolia Autonomous Region is characterized by arid and semi-arid agricultural ecosystems, limited water resources, and relatively high dependence on agricultural inputs such as irrigation, chemical fertilizers, diesel fuel, and plastic film mulching. Clarifying the temporal evolution of input-related carbon footprints in crop production is important for promoting agricultural low-carbon transformation while maintaining regional food security. Based on an input-based carbon footprint accounting framework with reference to life cycle assessment principles, this study quantified the carbon footprint of major crop production in Inner Mongolia from 2006 to 2023. Six major emission sources were considered: chemical fertilizers, pesticides, agricultural plastic film, diesel fuel, irrigation, and soil tillage. The results showed that agricultural input use experienced a transition from rapid expansion to partial stabilization. Fertilizer, pesticide, and diesel consumption increased during the early and middle stages of the study period and then stabilized or declined after the mid-2010s, whereas plastic film use continued to increase.The reported effective irrigated area increased sharply in 2022. This increase should be interpreted primarily as a statistical breakpoint caused by the official application of the Third National Land Survey results and the updated land-use classification system, rather than as a sudden one-year physical expansion of irrigation. The total carbon footprint increased from 448.88 × 10⁴ t CO₂-eq in 2006 to 720.07 × 10⁴ t CO₂-eq in 2023, representing an increase of 60.4%. Cropland tillage and chemical fertilizer application were the dominant emission sources, accounting for 38.2%–45.9% and 25.1%–31.4% of total emissions, respectively. Although the total carbon footprint and carbon footprint per unit planted area increased, the carbon footprint per unit agricultural output value decreased by 66.8%, indicating improved carbon-economic efficiency. However, this decline may also be influenced by market price changes and crop value structure. The findings suggest that future mitigation should focus on conservation tillage, precise fertilizer management, water-saving and energy-efficient irrigation, plastic film recycling or substitution, and improved agricultural carbon monitoring. This study provides a regional-scale reference for developing low-carbon agricultural pathways in arid and semi-arid farming systems.

## 1. Introduction

China’s “dual carbon” goals, namely carbon peaking and carbon neutrality, have made the low-carbon transformation of agriculture an important component of national sustainable development strategies [1]. Agriculture is a fundamental sector for ensuring food security, but it is also an important source of greenhouse gas emissions due to the use of fertilizers, pesticides, agricultural machinery, irrigation, and other production inputs [2]. The carbon footprint has been widely used to quantify direct and indirect greenhouse gas emissions associated with agricultural production systems and to support emission reduction policies and low-carbon management strategies [3]. However, agricultural production systems differ substantially across regions, and carbon emission characteristics in arid and semi-arid regions remain insufficiently understood compared with those in humid and semi-humid agricultural areas [4].

The Inner Mongolia Autonomous Region is an important grain-producing region in northern China and has typical arid and semi-arid agricultural characteristics [5]. The region also serves as an important ecological security barrier in northern China. Crop production in this region is constrained by limited precipitation, uneven seasonal water availability, and fragile ecosystems. As a result, agricultural production depends heavily on irrigation, plastic film mulching, mechanized operations, and chemical inputs. These characteristics distinguish the region from humid agricultural systems and make its crop production more sensitive to water–energy–carbon interactions [6,7]. In particular, irrigation agriculture in arid and semi-arid regions is often associated with considerable energy consumption, especially where groundwater pumping and electrically powered irrigation systems are widely used [8]. Therefore, identifying the temporal dynamics and dominant sources of agricultural carbon emissions in this region is important for designing regionally adapted low-carbon pathways.

Previous studies have used life cycle assessment, carbon emission coefficient methods, and carbon footprint indicators to evaluate agricultural emissions at national, provincial, and regional scales [9–13]. These studies have provided useful evidence on the relationship between agricultural inputs, crop production, and carbon emissions. However, many existing studies have focused on humid or semi-humid regions, while relatively few have examined long-term carbon footprint changes in arid and semi-arid farming systems [14,15]. In addition, some studies have emphasized total emissions but paid less attention to the differentiated behavior of multiple carbon intensity indicators, such as emissions per unit yield, per unit planted area, and per unit agricultural output value [16]. These indicators are important because total emissions, land-based pressure, physical production efficiency, and economic carbon efficiency may not change synchronously.

It should also be noted that regional agricultural carbon footprint accounting based on statistical data is different from a complete process-based life cycle assessment. A full LCA generally requires detailed life cycle inventory data, allocation procedures, life cycle impact assessment, uncertainty analysis, and often specialized LCA software. However, long-term regional statistical data often support only input-based carbon accounting for selected agricultural inputs. Therefore, it is necessary to clearly define the system boundary, functional units, included emission sources, and limitations of such studies to avoid overinterpretation.

To address these issues, this study takes major crop production in Inner Mongolia as the research object and analyzes the temporal evolution of input-related carbon footprints from 2006 to 2023. The specific objectives are:

(1) to quantify the input-related carbon footprint of crop production based on six major emission sources;
(2) to identify the dominant emission sources and changes in carbon footprint intensity;
(3) to analyze the relationship among agricultural inputs, planting structure, production output, and carbon footprint indicators;
(4) to propose low-carbon agricultural pathways suitable for arid and semi-arid farming conditions.

The results are expected to provide scientific support for agricultural carbon reduction, input optimization, and sustainable dryland agriculture development in northern China.

## 2. Materials and methods

### 2.1. Study area

The Inner Mongolia Autonomous Region is located in northern China and extends across a broad east–west ecological gradient. The region is characterized by extensive arid and semi-arid agricultural systems, limited precipitation, strong evapotranspiration, and uneven spatial and seasonal water availability. These natural conditions make crop production highly dependent on irrigation, soil moisture conservation, plastic film mulching, and mechanized field operations.

The region plays an important role in ensuring grain supply in northern China. Grain crops occupy the dominant position in the regional planting structure, while oil-bearing crops, sugar beet, vegetables, edible melons, forage crops, and other crops also contribute to agricultural production and economic output. Because of water scarcity and fragile ecosystems, the study area faces the dual challenge of maintaining agricultural productivity and reducing carbon emissions. Therefore, it provides a representative case for analyzing input-related carbon footprint dynamics and low-carbon transition pathways in arid and semi-arid agricultural regions.

### 2.2. Research system boundary and accounting framework

This study adopts an input-based carbon footprint accounting framework with reference to life cycle assessment principles, rather than a complete process-based LCA. The purpose of the study is to quantify the temporal evolution of input-related carbon emissions from crop production in the Inner Mongolia Autonomous Region from 2006 to 2023 based on long-term regional statistical data.

The system boundary covers the main agricultural inputs and field management activities that can be consistently obtained from official statistical sources over the study period, including chemical fertilizers, pesticides, agricultural plastic film, diesel fuel, irrigation, and soil tillage. These sources represent the major measurable input-related emission categories of regional crop production. Chemical fertilizers, pesticides, and plastic film mainly reflect indirect emissions associated with the production and use of agricultural materials; diesel fuel represents fossil-energy-related emissions from agricultural machinery operations; irrigation represents energy-related emissions associated with effective irrigated area; and soil tillage represents carbon emissions associated with cultivated land disturbance.

The life cycle inventory used in this study includes annual activity data for fertilizer application, pesticide use, plastic film use, agricultural diesel consumption, effective irrigated area, and cultivated or sown area. The functional units used for evaluation include: total carbon footprint of agricultural inputs, expressed as 10⁴ t CO₂-eq; carbon footprint per unit crop yield, expressed as t CO₂-eq·t ⁻ ¹; carbon footprint per unit planted area, expressed as t CO₂-eq·ha ⁻ ¹; and carbon footprint per unit agricultural output value, expressed as t CO₂-eq·10 ⁻ ⁴ yuan.

It should be noted that this study estimates the gross input-related carbon footprint of crop production and does not represent a complete agricultural greenhouse gas inventory. Due to limitations in long-term statistical data, direct field emissions such as N₂O from fertilizer application and CH₄ from rice paddies, as well as emissions from crop residue management, harvesting, threshing, weed control, plastic film end-of-life treatment, and soil carbon sequestration, were not separately quantified. Therefore, the results should be interpreted as regional-scale input-related carbon accounting rather than full life-cycle environmental assessment.

### 2.3. Data sources

The data on effective irrigated area, fertilizer application, pesticide use, agricultural plastic film use, agricultural diesel consumption, planting areas of major crops, crop yields, and total agricultural output value in Inner Mongolia from 2006 to 2023 were obtained from the annual issues of the *Inner Mongolia Statistical Yearbook* and the National Bureau of Statistics of China. The crops covered in the analysis include major food crops cultivated in the region, such as wheat, corn, rice, soybeans, and tubers; cash crops, including oil-bearing crops, sugar beets, tobacco leaves, fiber crops, vegetables, and edible melons; and other crops such as forage crops.

It should be noted that the effective irrigated area series shows a statistical breakpoint in 2022. This breakpoint is mainly related to the official application of the results of the Third National Land Survey and the corresponding adjustment of land-use classification and statistical coverage [17,18]. Compared with the previous statistical basis, the Third National Land Survey re-identified cultivated land and irrigated farmland using updated land survey, remote sensing, field verification, and quality-control procedures [17,18]. Therefore, the sharp increase in effective irrigated area in 2022 should not be interpreted simply as a one-year physical expansion of irrigation, but as a statistical break adjustment supported by updated land survey and land change survey data [17,18]. Additional official water resources bulletins and farmland construction documents were also used to support the interpretation and external consistency check of the 2022 irrigation-area change [19–22].

To ensure the comparability of long-term data, this study used indicators with continuous annual records and consistent statistical definitions as far as possible. Nevertheless, possible changes in statistical reporting scope or data collection methods over the study period may introduce uncertainty, especially for indicators such as effective irrigated area. This issue is considered in the interpretation and limitations of the study.

### 2.4. Calculation methods

To assess the carbon footprints associated with crop production inputs in Inner Mongolia from 2006 to 2023, four indicators were employed: the total carbon footprint of agricultural inputs (CF), the carbon footprint per unit crop yield (CF_Y_), the carbon footprint per unit planted area (CF_A_), and the carbon footprint per unit agricultural output value (CF_E_). The calculation formulas are as follows:


$$\begin{array}{c} CF=\sum_{\text{i}=1}^{\text{6}}E_{\text{i}}\times C_{\text{i}} \end{array}$$ (1)

$$\begin{array}{c} CF_{Y}=\frac{CF}{Y} \end{array}$$ (2)

$$\begin{array}{c} CF_{A}=\frac{CF}{A} \end{array}$$ (3)

$$\begin{array}{c} CF_{E}=\frac{CF}{E} \end{array}$$ (4)

In the formulas, CF denotes the carbon footprint of agricultural inputs (10⁴ t CO₂-eq); E_i_ represents the input quantity of the i-th carbon source, where i = 1, 2,..., 6; and C_i_ refers to the carbon emission coefficient associated with the i-th carbon source. CF_Y_ represents the carbon footprint per unit crop yield (t CO₂-eq·t ⁻ ¹); CF_A_ represents the carbon footprint per unit planted area (t CO₂-eq·ha ⁻ ¹); and CF_E_ represents the carbon footprint per unit agricultural output value (t CO₂-eq·10 ⁻ ⁴ yuan). Y denotes crop yield (10⁴ t); A denotes crop planting area (10³ ha), calculated as the sum of the planting areas of major crops in the region; and E denotes total annual agricultural output value (10⁸ yuan).

The carbon emission coefficients were selected from IPCC-related accounting principles and previous domestic and international studies. All coefficients were converted to a consistent CO₂-equivalent basis and matched with the corresponding statistical activity data before calculation. Because consistent annual data on regional electricity mix, fertilizer type structure, soil type, tillage depth, irrigation pumping depth, and machinery operation intensity were unavailable, fixed coefficients were used to maintain temporal comparability over the 2006–2023 period. The coefficients used in this study are shown in Table 1.

**Table 1:** Carbon emission coefficients of different carbon sources in crop farming.

Carbon source	Carbon emission coefficient	Reference source
Chemical fertilizer	0.89(t CO_2_-eq·t ^− 1^）	[23]
Pesticide	4.93(t CO_2_-eq·t ^− 1^）	[23]
Plastic film	5.18(t CO_2_-eq·t ^− 1^）	[23]
Irrigation	266.48(t CO_2_-eq·10^3^ha ^− 1^)	[24]
Diesel	0.59(t CO_2_-eq·t ^− 1^)	[25]
Soil tillage	312.60(t CO_2_-eq·10^3^ha ^− 1^)	[24]

**Note:** The coefficients were selected from IPCC-related accounting principles and previous domestic and international studies and were used for regional-scale input-related carbon accounting. They were not dynamically adjusted by annual electricity mix, fertilizer type, soil type, or tillage intensity due to data limitations.

### 2.5. Statistical analysis

Data were organized and calculated using Microsoft Excel 2016, and figures were generated using Origin 2024. The analysis focused on temporal trends, phased changes, source contributions, and carbon footprint intensity indicators. No GIS-based spatial analysis or complete process-based LCA software modeling was conducted in this study.

## 3. Results and analysis

### 3.1. Evolution of agricultural inputs for crop production

**Fig 1:**
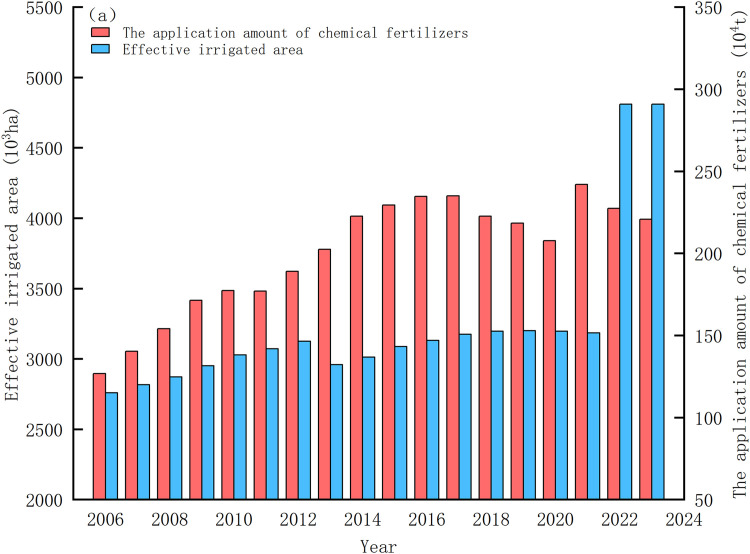
Year-on-year changes in fertilizer application and effective irrigated area for crop production in the Inner Mongolia Autonomous Region from 2006 to 2023.

**Fig 2:**
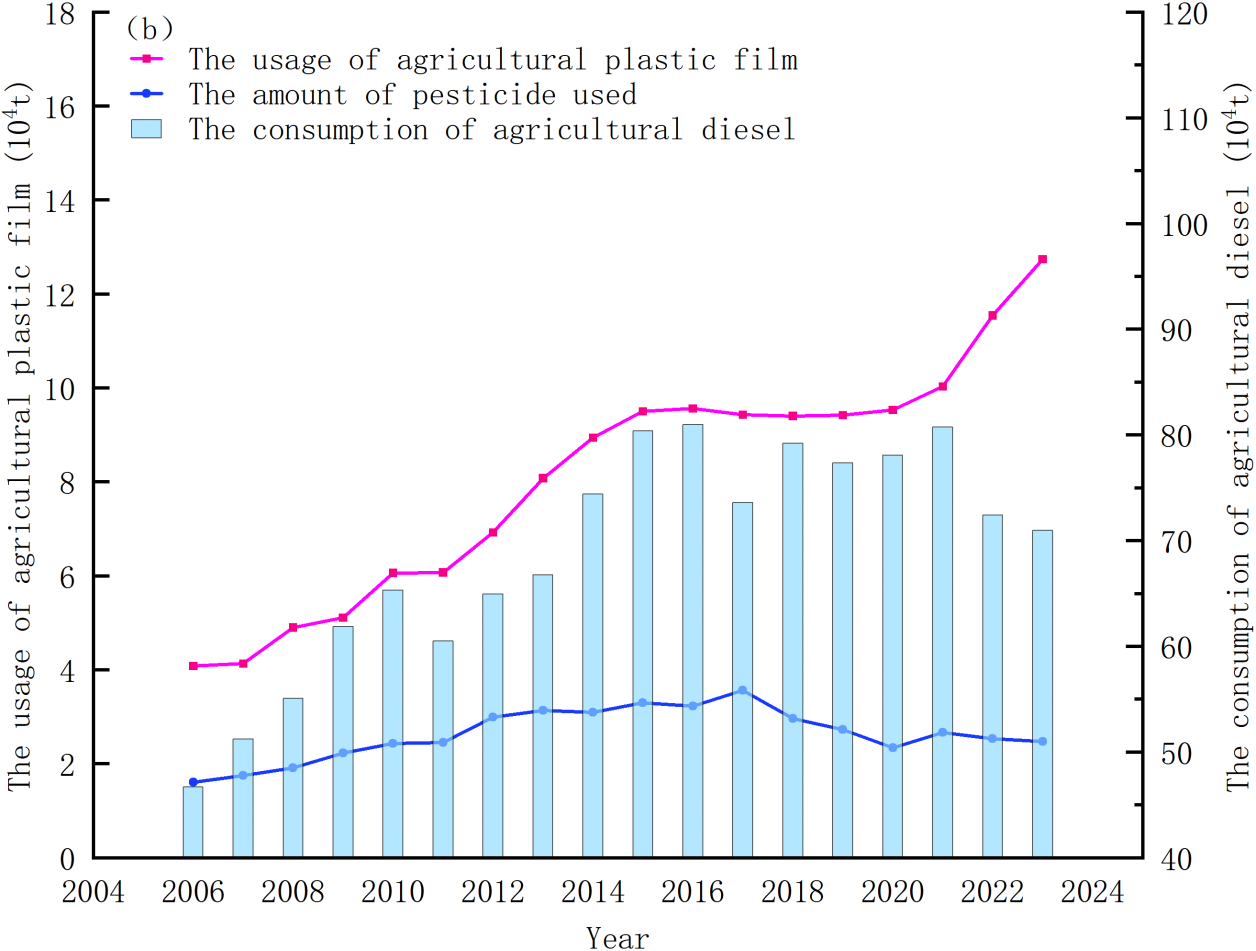
Year-on-year changes in agricultural plastic film use, pesticide application, and agricultural diesel consumption for crop production in the Inner Mongolia Autonomous Region from 2006 to 2023.

The evolution of agricultural inputs in the study area from 2006 to 2023 indicates a transition from input-driven expansion to regulated input optimization, although different input categories showed distinct trajectories. As shown in Figs 1 and 2, fertilizer application, pesticide use, and diesel consumption generally increased during the first half of the study period and then stabilized or declined after the mid-2010s. In contrast, plastic film use maintained a persistent upward trend, while the effective irrigated area showed a marked structural increase in 2022.

Fertilizer application increased from 126.70 × 10⁴ t in 2006 to 235.04 × 10⁴ t in 2017 and reached 241.90 × 10⁴ t in 2021, reflecting the strong dependence of crop yield improvement on nutrient inputs during the expansion stage of agricultural production. After 2021, fertilizer application declined to 220.80 × 10⁴ t in 2023. This decline suggests that the growth of agricultural production was no longer solely dependent on continuous fertilizer expansion, which may be associated with improved fertilizer management, soil testing-based fertilization, and policy guidance for reducing excessive chemical input. Pesticide use showed a similar pattern, increasing from 1.61 × 10⁴ t in 2006 to a peak of 3.56 × 10⁴ t in 2017, followed by a decline to 2.47 × 10⁴ t in 2023. The simultaneous reduction in fertilizer and pesticide use after 2017 indicates a gradual shift from quantity-oriented input growth toward more regulated input management.

Agricultural diesel consumption also exhibited a phased pattern. It increased from 46.69 × 10⁴ t in 2006 to 81.00 × 10⁴ t in 2016, which was closely related to the expansion of mechanized agricultural operations. After 2016, diesel consumption fluctuated downward and reached 70.96 × 10⁴ t in 2023. This suggests that the contribution of mechanization to agricultural expansion remained important, but its growth intensity weakened in the later stage. Unlike fertilizer, pesticide, and diesel, plastic film use increased continuously from 4.08 × 10⁴ t in 2006 to 12.74 × 10⁴ t in 2023. This persistent increase reflects the structural demand for soil moisture conservation in arid and semi-arid farming systems. Therefore, plastic film use should not be interpreted simply as an input increase, but as a region-specific adaptation to water scarcity and unstable precipitation.

The effective irrigated area remained relatively stable between 2006 and 2021, increasing from 2758.10 × 10³ ha to 3184.00 × 10³ ha. However, it increased sharply to 4809.00 × 10³ ha in 2022 and remained at this level in 2023. This abrupt increase should be interpreted as a statistical breakpoint rather than as a purely physical one-year expansion of irrigation. The main reason is that the results of the Third National Land Survey and the updated land-use classification system were officially incorporated into land and agricultural statistics around this period [17,18]. Compared with the previous statistical basis, the updated survey system more comprehensively identified cultivated land with irrigation facilities or stable irrigation conditions. As a result, part of the irrigated farmland that had not been fully reflected in earlier statistical series was reclassified or newly incorporated into the effective irrigated area category [17,18].

Therefore, the 2022 increase reflects the combined effect of statistical reclassification and accumulated irrigation infrastructure improvement, including high-standard farmland construction and water-saving irrigation projects [18,21,22]. Because irrigation emissions in this study were estimated based on effective irrigated area, this statistical breakpoint directly increased the calculated irrigation-related carbon footprint in 2022 and 2023. Thus, the irrigation-related results after 2022 should be interpreted cautiously and should not be regarded simply as evidence of a sudden increase in groundwater pumping or irrigation water use.

Overall, the agricultural input system of the study area showed three major patterns: first, fertilizer, pesticide, and diesel use shifted from rapid growth to stabilization or decline; second, plastic film use continued to rise due to the moisture-conservation requirements of dryland and irrigated agriculture; and third, the sharp expansion of effective irrigation area after 2022 became a new driver of calculated carbon footprint growth, although this change was strongly affected by the statistical breakpoint. These patterns suggest that future emission reduction should focus not only on reducing chemical inputs but also on improving irrigation energy efficiency and plastic film management.

### 3.2. Changes in the planting structure of major crops

**Fig 3:**
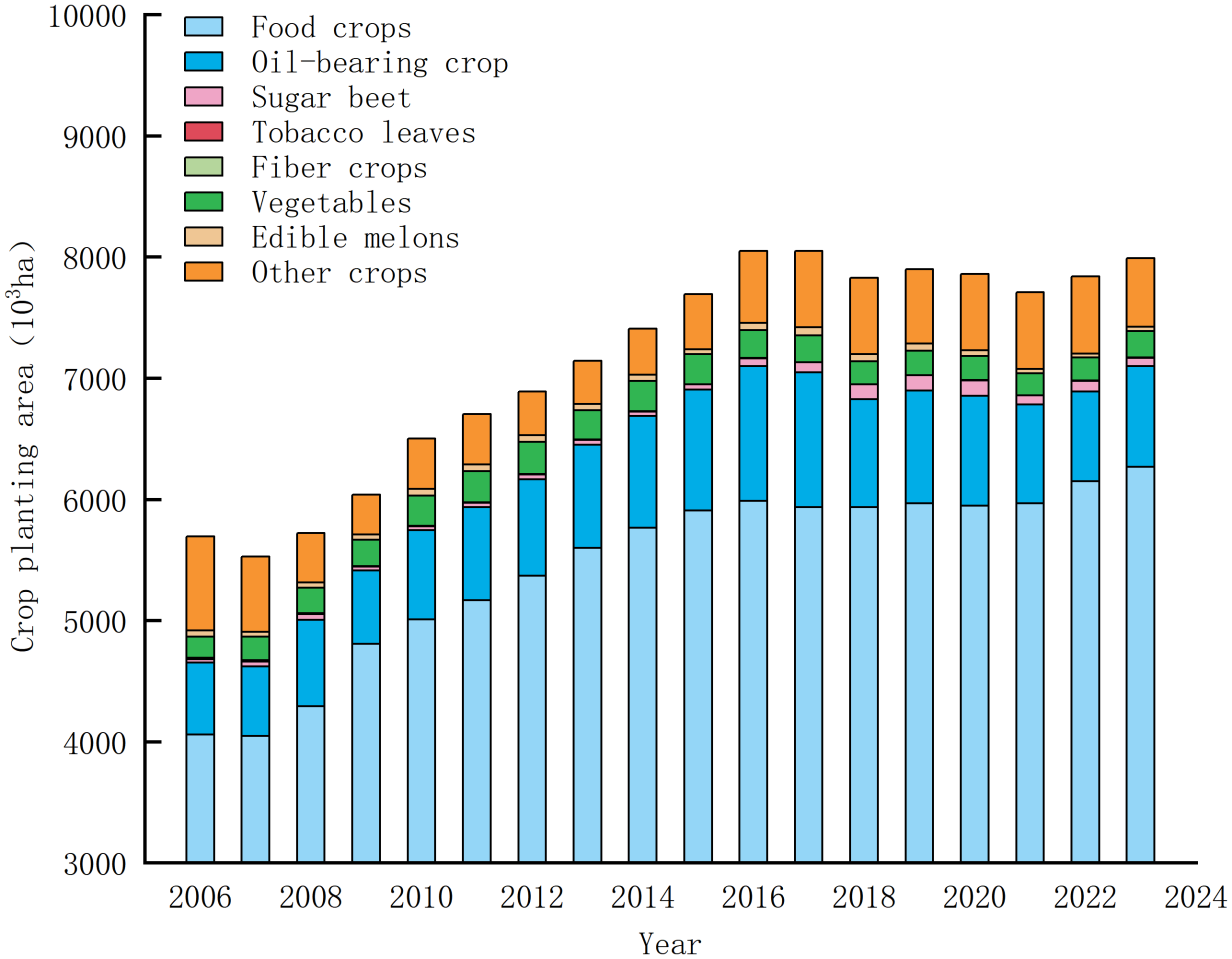
Cultivated areas of different crop types in the Inner Mongolia Autonomous Region from 2006 to 2023. Grain crops were dominant throughout the study period, while oil-bearing crops, sugar beet, vegetables, edible melons, and other crops showed different degrees of fluctuation.

The planting structure of the study area was dominated by grain crops throughout the study period, while cash crops and other crops showed more pronounced fluctuations. As shown in Table 2 and Fig 3, grain crops consistently accounted for the largest share of the total sown area, ranging from 61.6% to 71.6%. The cultivated area of grain crops increased from 4059 × 10³ ha in 2006–6266 × 10³ ha in 2023, representing an increase of 54.4%. This expansion confirms the central role of grain production in the regional agricultural system and reflects the strategic function of the study area in maintaining food security in northern China.

**Table 2:** Main Crop Yields and Total Agricultural Output Value of Inner Mongolia Autonomous Region from 2006 to 2023.

Year	Food crops（10^4^t）	Oil-bearing crop（10^4^t）	Sugar beet（10^4^t）	Tobacco leaves（10^4^t）	Fiber crops（10^4^t）	Vegetables（10^4^t）	Edible melons（10^4^t）	Total output value of agriculture（10^8^yuan）
2006	1692.8	101.1	105.5	2.6	1.7	1171.4	190.8	542.2
2007	1667.1	96.3	171.5	1.5	1.4	1045.4	154.9	623.1
2008	1990.0	135.1	192.8	1.4	2.8	1050.9	176.8	722.8
2009	2050.6	122.7	103.7	1.2	1.0	1083.4	152.7	741.4
2010	2246.4	138.1	145.1	1.5	0.1	1326.1	220.8	916.1
2011	2451.6	149.0	141.4	1.5	0	1388.6	207.8	1080.9
2012	2606.5	142.2	149.4	1.4	0	1354.8	198.6	1202.8
2013	2957.6	161.8	161.3	1.3	0	1300.1	165.7	1368.9
2014	3008.1	180.7	143.9	1.1	0	1318.9	185.3	1457.9
2015	3162.3	206.3	200.5	1.2	0	1284.9	158.3	1474.5
2016	3123.8	228.8	268.4	0.9	0.2	1251.8	242.2	1477.6
2017	3095.4	240.7	344.3	0.6	0.7	1111.3	267.5	1434.7
2018	3379.7	201.5	515.9	0.6	0.2	1006.5	225.2	1512.5
2019	3431.9	228.7	629.6	0.4	0.3	1090.8	230.2	1606.3
2020	3419.1	217.3	620.7	0.5	0.3	1075.1	191.9	1699.0
2021	3577.90	213.9	362.0	0.3	0.1	993.7	136.6	1879.6
2022	3686.5	170.0	385.1	0.4	0.3	1012.9	119.0	2208.5
2023	3780.0	206.2	304.8	0.4	0.5	1097.1	152.4	2288.0

**Note:** Annual yields of major crops and total agricultural output value of the Inner Mongolia Autonomous Region from 2006 to 2023. The table reflects the temporal dynamics of crop production scale and agricultural economic output.

The expansion of grain crop area was accompanied by a substantial increase in grain output. Grain production increased from 1692.8 × 10⁴ t in 2006 to 3780.0 × 10⁴ t in 2023. The rate of increase in grain production was higher than that of grain sown area, indicating that yield improvement also contributed substantially to output growth. This suggests that the regional crop production system experienced not only area expansion but also productivity improvement. However, because grain crops occupy the dominant share of cultivated land, changes in grain production are likely to exert a disproportionate influence on total agricultural carbon emissions, especially through tillage, fertilizer application, diesel use, and irrigation demand.

Compared with grain crops, oil-bearing crops showed a more fluctuating trajectory. Their cultivated area increased from 592 × 10³ ha in 2006–1113 × 10³ ha in 2017, followed by a decline to 834 × 10³ ha in 2023. This pattern indicates that oil-bearing crop production was more sensitive to market demand, planting structure adjustment, and regional cropping decisions. Sugar beet area also experienced strong fluctuations, rising from 30 × 10³ ha in 2006 to a peak of 127 × 10³ ha in 2019 before declining to 66 × 10³ ha in 2023. Vegetables maintained a relatively small but stable share of the sown area, fluctuating between 172 × 10³ ha and 267 × 10³ ha during the study period. Tobacco leaves, fiber crops, and edible melons accounted for relatively small proportions and therefore had limited direct influence on the total carbon footprint at the regional scale.

The increase in total agricultural output value from 542.2 × 10⁸ yuan in 2006 to 2288.0 × 10⁸ yuan in 2023 indicates that agricultural economic output improved substantially. However, this increase should be interpreted cautiously when evaluating carbon efficiency, because the carbon footprint per unit output value may be affected not only by environmental efficiency but also by changes in agricultural prices, crop composition, and market value. Therefore, the decline in carbon footprint intensity per output value reflects an improvement in agricultural eco-economic performance, but it should not be regarded as equivalent to a proportional reduction in physical emissions.

In summary, the planting structure was characterized by the long-term dominance of grain crops, the fluctuating adjustment of cash crops, and the continuous increase in agricultural output value. These structural features explain why total emissions continued to rise even when some input intensities declined: the expansion of grain-oriented production increased the total demand for land preparation, fertilization, mechanization, and irrigation.

### 3.3. Temporal evolution of the carbon footprint of agricultural production

**Fig 4:**
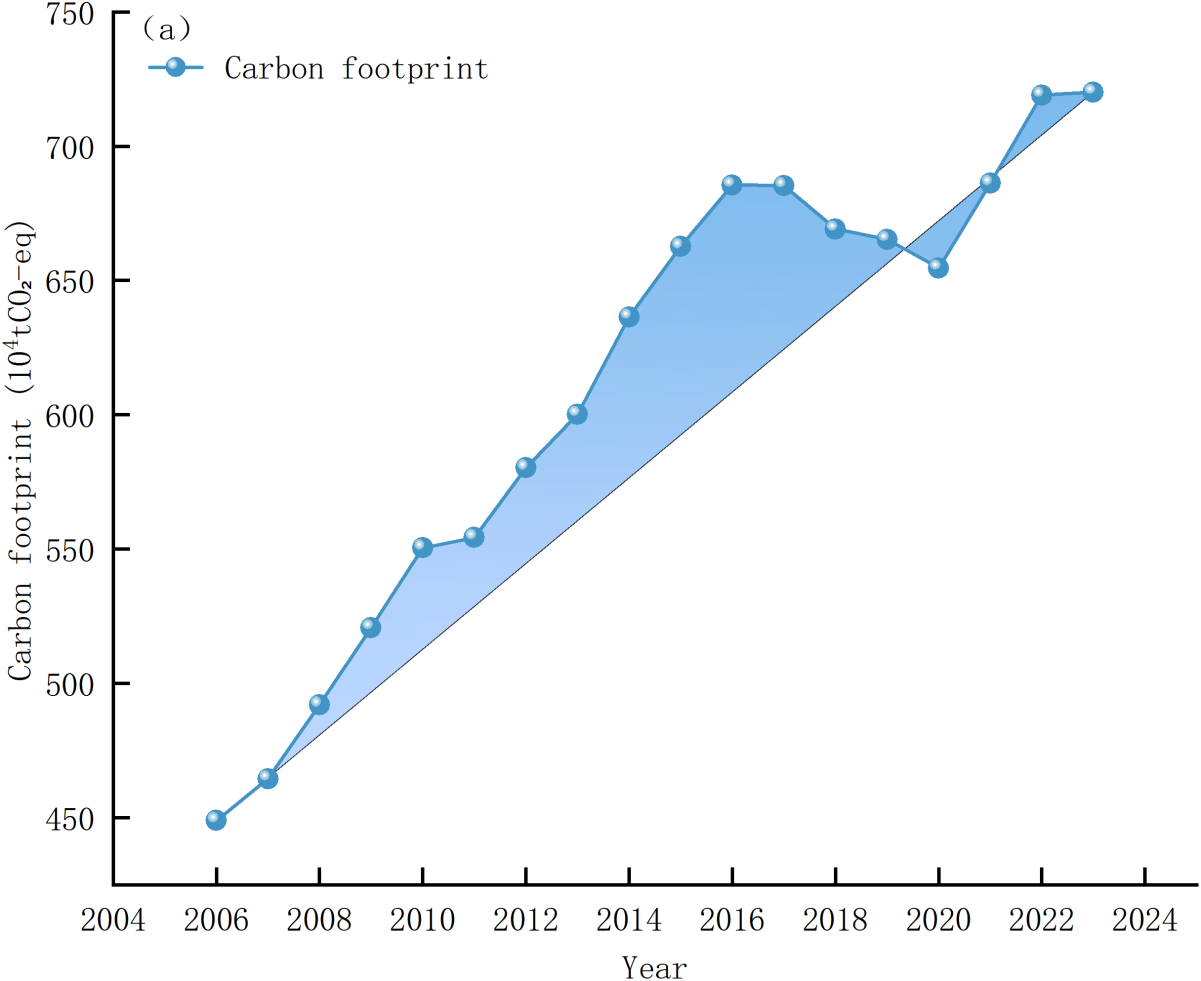
Temporal changes in the total carbon footprint of agricultural production in the Inner Mongolia Autonomous Region from 2006 to 2023.

**Fig 5:**
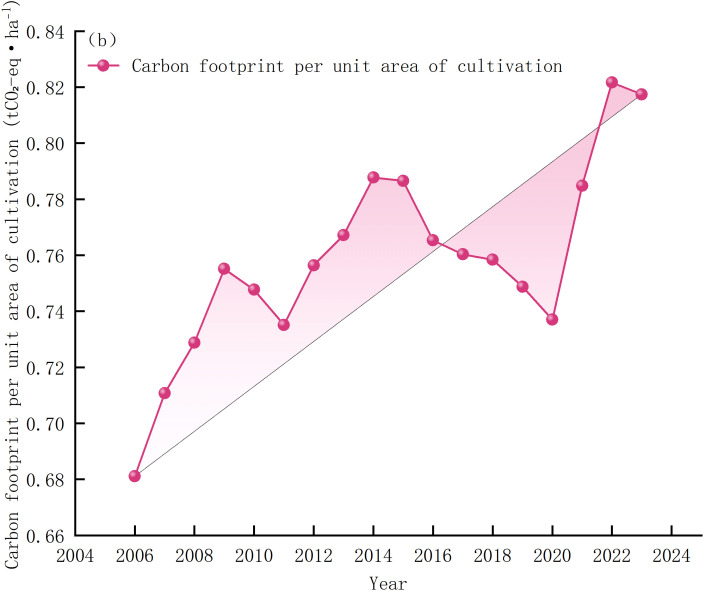
Temporal changes in the carbon footprint per unit crop yield in the Inner Mongolia Autonomous Region from 2006 to 2023.

**Fig 6:**
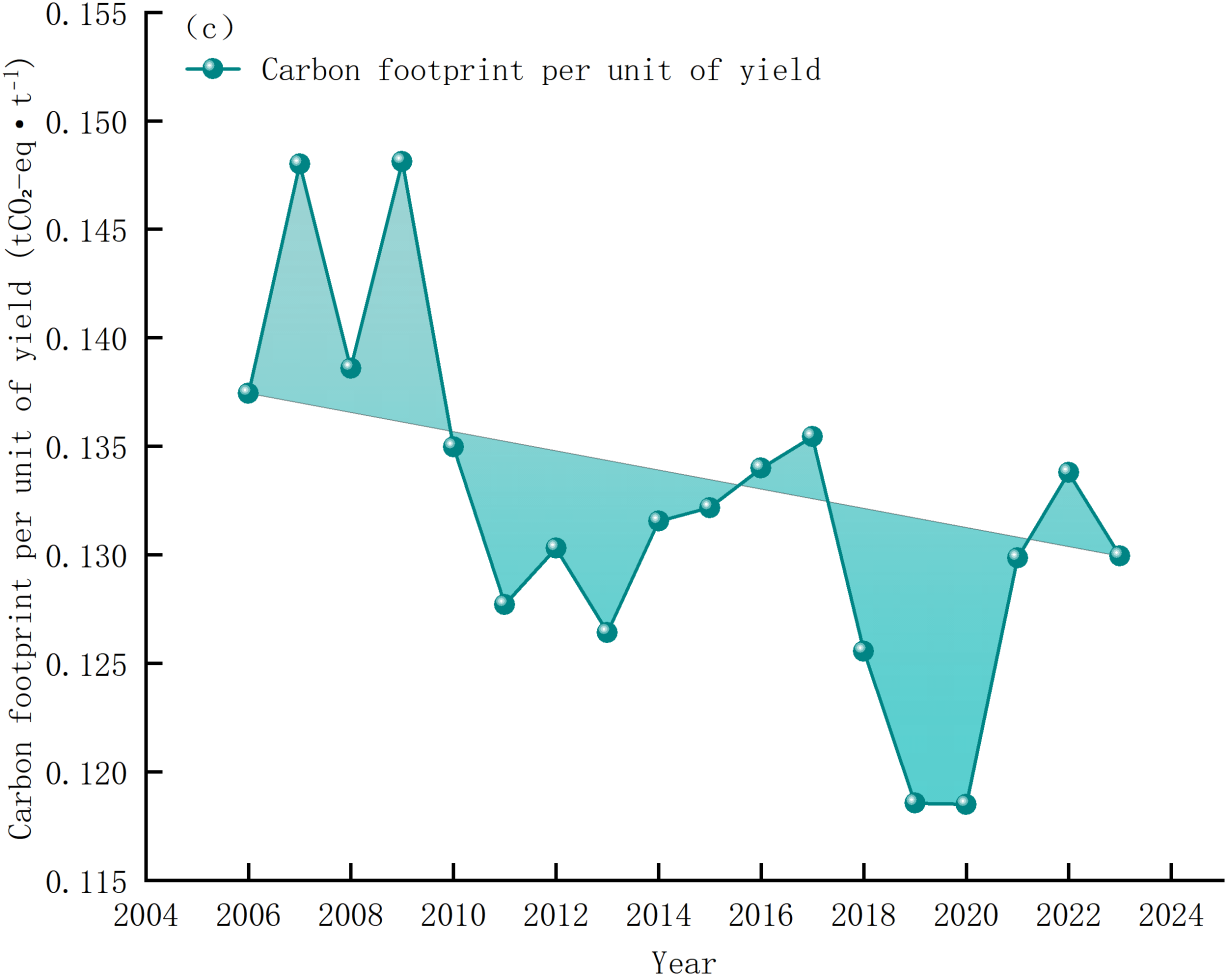
Temporal changes in the carbon footprint per unit planted area in the Inner Mongolia Autonomous Region from 2006 to 2023.

**Fig 7:**
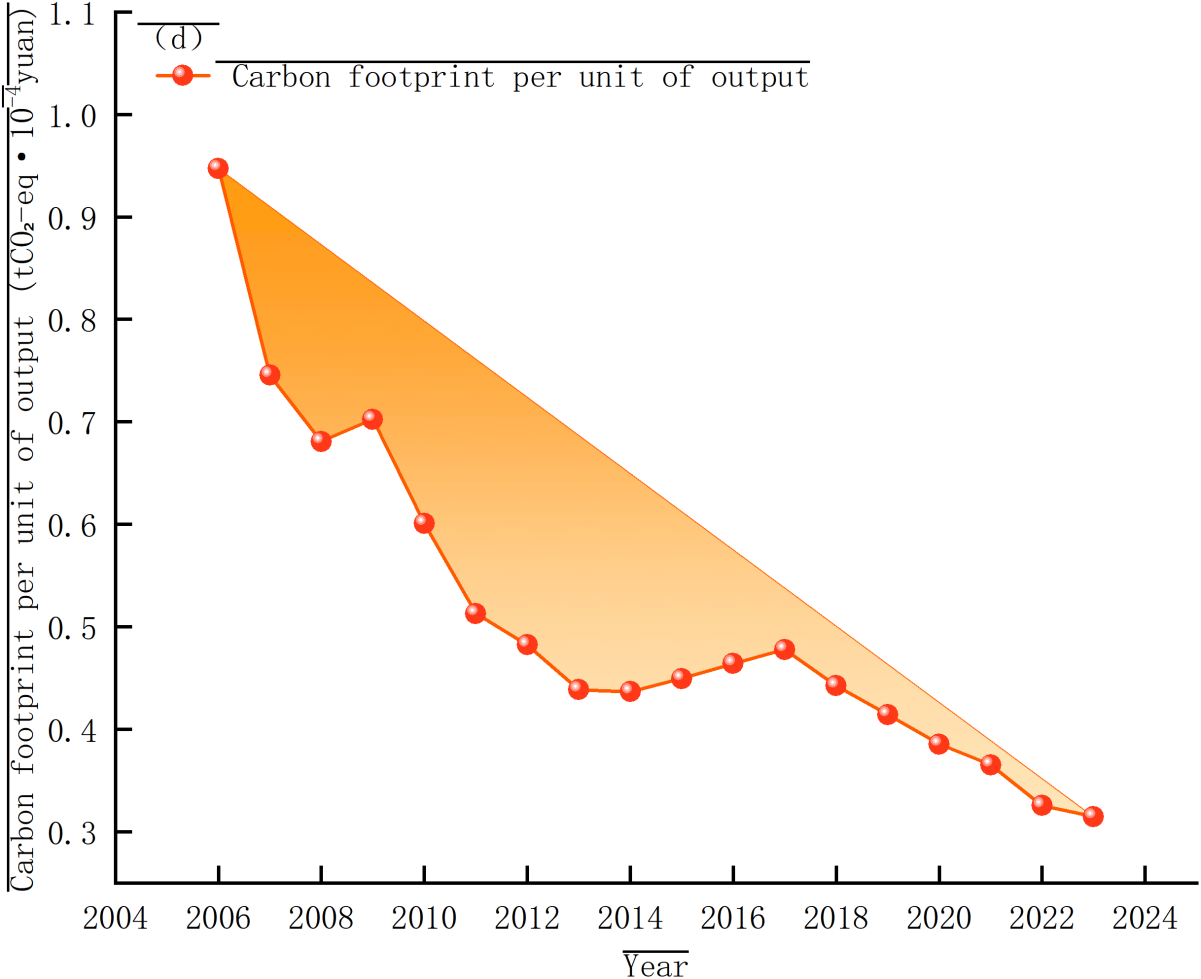
Temporal changes in the carbon footprint per unit agricultural output value in the Inner Mongolia Autonomous Region from 2006 to 2023.

The carbon footprint indicators showed differentiated trends from 2006 to 2023, indicating that agricultural production scale, land-use intensity, production efficiency, and economic output did not evolve synchronously. As shown in Figs 4–7, the total carbon footprint of agricultural inputs increased overall, while the carbon footprint per unit output value decreased continuously. This divergence suggests a partial decoupling between agricultural economic growth and carbon emission intensity.

The total carbon footprint increased from 448.88 × 10⁴ t CO₂-eq in 2006 to 720.07 × 10⁴ t CO₂-eq in 2023, representing a 60.4% increase. The increase was not linear. During 2006–2017, the total carbon footprint rose rapidly, mainly due to the expansion of fertilizer application, diesel consumption, plastic film use, and cultivated area. After 2017, the growth rate slowed, and the carbon footprint remained relatively stable from 2018 to 2021. This stabilization corresponded with the reduction or stabilization of fertilizer, pesticide, and diesel inputs. However, the total carbon footprint increased again in 2022 and 2023, mainly because of the reported increase in effective irrigated area and the associated rise in irrigation-related emissions. As noted above, this post-2022 change should be interpreted cautiously because of the statistical breakpoint in the effective irrigated area series.

The carbon footprint per unit crop yield showed a different pattern. It increased from 0.13745 t CO₂-eq·t ⁻ ¹ in 2006 to 0.14813 t CO₂-eq·t ⁻ ¹ in 2009, indicating that the early expansion of crop production was accompanied by relatively high carbon input per unit output. After 2009, this indicator generally declined and reached 0.11849 t CO₂-eq·t ⁻ ¹ in 2020. The decline suggests that yield improvement gradually offset part of the emission increase caused by agricultural input expansion. After 2020, the indicator fluctuated slightly, reaching 0.12994 t CO₂-eq·t ⁻ ¹ in 2023. This recent rebound may be affected by the renewed increase in calculated irrigation-related emissions and continued plastic film use.

The carbon footprint per unit planted area increased from 0.68116 t CO₂-eq·ha ⁻ ¹ in 2006 to 0.81742 t CO₂-eq·ha ⁻ ¹ in 2023, with the highest value of 0.82166 t CO₂-eq·ha ⁻ ¹ occurring in 2022. This result indicates that although agricultural production efficiency improved in terms of output and economic value, the emission pressure per unit land area increased. In other words, land-use intensity became more carbon-intensive over time. This is particularly important for arid and semi-arid agricultural systems, where higher productivity often depends on irrigation, plastic film mulching, mechanization, and fertilizer inputs.

The carbon footprint per unit agricultural output value showed the most significant decline, decreasing from 0.94723 t CO₂-eq·10 ⁻ ⁴ yuan in 2006 to 0.31471 t CO₂-eq·10 ⁻ ⁴ yuan in 2023, a reduction of 66.8%. This indicates that the carbon emission intensity of agricultural economic output declined substantially. However, because total agricultural output value is influenced by price changes, market structure, and crop value composition, this decline should be interpreted as an improvement in carbon-economic efficiency rather than direct evidence of equivalent physical emission reduction. Therefore, the simultaneous increase in total emissions and decline in output-value-based intensity reveals a dual process: agricultural production became economically more carbon-efficient, but the absolute carbon burden of crop production continued to increase.

Overall, the four indicators together reveal a more nuanced picture than a single carbon footprint metric. Total CF and CF_A_ increased, indicating rising absolute emissions and land-based emission pressure. CF_Y_ declined after the early stage, suggesting improved physical production efficiency. CF_E_ declined continuously, reflecting improved economic carbon efficiency. These results imply that future low-carbon transformation should not focus only on reducing total inputs, but should also enhance yield stability, improve irrigation energy efficiency, and reduce land-based emission intensity.

### 3.4. Contribution of different carbon sources to the total carbon footprint

**Fig 8:**
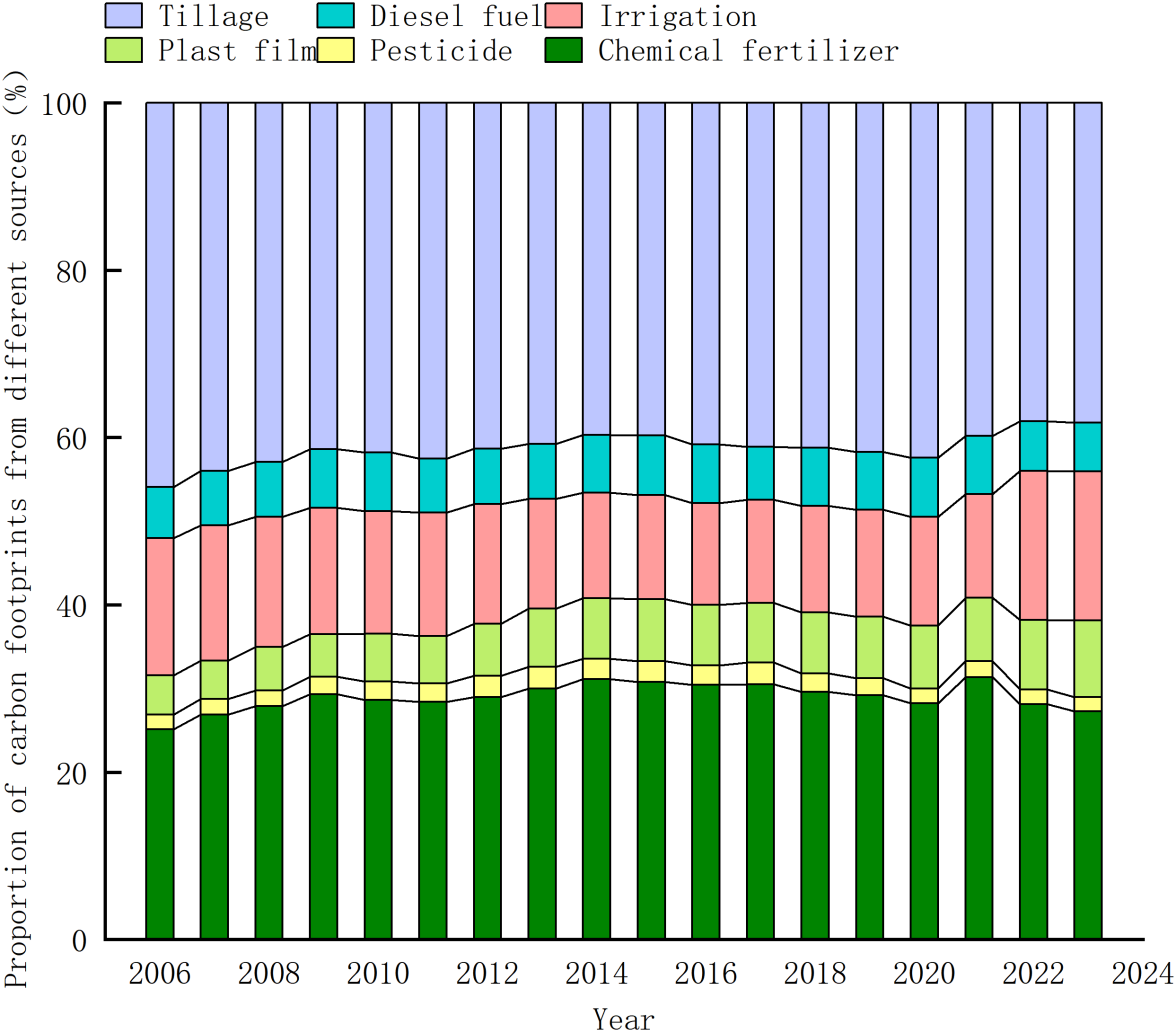
Contribution of different carbon sources to the total carbon footprint of agricultural production in the Inner Mongolia Autonomous Region from 2006 to 2023. Cropland tillage and chemical fertilizer application were the dominant sources, while irrigation and plastic film showed increasing importance in recent years.

The source-specific carbon footprint results show that cropland tillage and fertilizer application were the two dominant contributors to agricultural carbon emissions during the study period. As shown in Fig 8, the contribution of cropland tillage ranged from 38.2% to 45.9%, while that of chemical fertilizer ranged from 25.1% to 31.4%. Together, these two sources accounted for the majority of the total carbon footprint, indicating that land-use scale and nutrient input intensity were the fundamental determinants of agricultural emissions in the study area.

The carbon footprint from cropland tillage increased from 206.00 × 10⁴ t CO₂-eq in 2006 to 275.37 × 10⁴ t CO₂-eq in 2023. Although its proportional contribution declined over time, it remained the largest emission source throughout the study period. This suggests that the expansion of cultivated area and the persistence of conventional tillage practices continued to generate substantial carbon pressure. The declining share of tillage emissions does not mean that tillage-related emissions became unimportant; rather, it reflects the faster growth of other sources, especially irrigation and plastic film in the later period.

Chemical fertilizer emissions increased from 112.76 × 10⁴ t CO₂-eq in 2006 to a peak of 215.29 × 10⁴ t CO₂-eq in 2021, before declining to 196.51 × 10⁴ t CO₂-eq in 2023. This trend corresponds closely to fertilizer application changes and indicates that fertilizer management remains one of the most effective entry points for emission reduction. The reduction after 2021 suggests that fertilizer control measures may have begun to moderate fertilizer-related emissions. Nevertheless, fertilizer remained the second-largest carbon source, showing that further improvement in nutrient use efficiency is still necessary.

Irrigation-related emissions exhibited the most prominent structural change. From 2006 to 2021, irrigation emissions remained within a relatively narrow range, increasing slightly from 73.50 × 10⁴ t CO₂-eq to 84.85 × 10⁴ t CO₂-eq. However, they rose sharply to 128.15 × 10⁴ t CO₂-eq in 2022 and remained at this level in 2023. This change was directly associated with the increase in effective irrigated area to 4809.00 × 10³ ha. Nevertheless, this increase should not be interpreted as a sudden one-year expansion of groundwater pumping. Rather, it was mainly caused by a statistical breakpoint associated with the application of the Third National Land Survey results and the updated classification of irrigated farmland [17,18].

External evidence also supports this interpretation. Water resources statistics indicate that agricultural water use increased only moderately in 2022, while irrigation water-use efficiency remained within a reasonable range [19,20]. This suggests that the increase in effective irrigated area was largely related to the re-identification and reclassification of existing irrigated farmland, together with accumulated improvements in irrigation infrastructure, rather than an unconstrained expansion of water consumption [17–22]. Therefore, irrigation-related carbon emissions after 2022 should be interpreted cautiously. Despite this statistical break, the result still indicates that irrigation is becoming an increasingly important component of agricultural carbon accounting in water-limited regions. Future mitigation should therefore focus on water-saving irrigation, energy-efficient pumping systems, and more precise monitoring of irrigation energy use.

Plastic film emissions increased continuously from 21.13 × 10⁴ t CO₂-eq in 2006 to 65.99 × 10⁴ t CO₂-eq in 2023. Although its share in total emissions was lower than those of tillage and fertilizer, its continuous increase deserves attention. Plastic film mulching is closely linked to soil moisture conservation, temperature regulation, and crop establishment in arid and semi-arid regions. Therefore, reducing plastic film-related emissions requires technological substitution and recycling governance rather than simple reduction in use. Biodegradable film, thicker recyclable film, and “one film for two years” practices may provide feasible mitigation pathways if their agronomic performance and environmental trade-offs are further validated.

Diesel-related emissions increased from 27.55 × 10⁴ t CO₂-eq in 2006 to 47.79 × 10⁴ t CO₂-eq in 2016, followed by a decline to 41.87 × 10⁴ t CO₂-eq in 2023. This indicates that mechanization-related emissions grew during the agricultural expansion stage but stabilized in the later period. Pesticide emissions remained the smallest contributor, increasing from 7.94 × 10⁴ t CO₂-eq in 2006 to 17.55 × 10⁴ t CO₂-eq in 2017 and then declining to 12.18 × 10⁴ t CO₂-eq in 2023.

In summary, the source contribution analysis identifies three priority areas for mitigation: reducing tillage intensity and improving soil management, optimizing fertilizer application, and improving irrigation energy efficiency. In addition, the continuous growth of plastic film emissions indicates that material substitution and recycling systems should be integrated into regional low-carbon agricultural policies.

### 3.5. Carbon footprint intensity of different emission sources

The carbon footprint intensity per unit cultivated area further reveals the changing emission pressure of different agricultural inputs. As shown in Table 3, the total intensity increased from 0.68116 t CO₂-eq·ha ⁻ ¹ in 2006 to 0.81742 t CO₂-eq·ha ⁻ ¹ in 2023, indicating that the emission pressure per unit land area increased despite improvements in output-based and value-based carbon efficiency. In the revised Table 3, soil tillage intensity is listed separately, and the total intensity represents the sum of chemical fertilizer, pesticide, plastic film, irrigation, diesel, and soil tillage.

**Table 3:** Emission intensity of major agricultural input-related carbon sources in crop production in the Inner Mongolia Autonomous Region from 2006 to 2023 (t CO₂-eq·ha ⁻ ¹).

Year	Chemical Fertilizer	Pesticide	Plastic Film	Irrigation	Diesel	Soil Tillage	Total
2006	0.1711	0.0120	0.0321	0.1115	0.0418	0.3126	0.6812
2007	0.1911	0.0132	0.0327	0.1149	0.0463	0.3126	0.7107
2008	0.2032	0.0139	0.0376	0.1133	0.0481	0.3126	0.7288
2009	0.2212	0.0159	0.0384	0.1140	0.0530	0.3126	0.7551
2010	0.2143	0.0163	0.0426	0.1096	0.0523	0.3126	0.7477
2011	0.2089	0.0160	0.0417	0.1086	0.0473	0.3126	0.7351
2012	0.2193	0.0192	0.0467	0.1086	0.0500	0.3126	0.7564
2013	0.2303	0.0197	0.0535	0.1008	0.0504	0.3126	0.7672
2014	0.2453	0.0189	0.0573	0.0993	0.0543	0.3126	0.7878
2015	0.2423	0.0193	0.0584	0.0976	0.0563	0.3126	0.7866
2016	0.2331	0.0178	0.0553	0.0932	0.0534	0.3126	0.7653
2017	0.2321	0.0195	0.0542	0.0939	0.0482	0.3126	0.7604
2018	0.2246	0.0165	0.0552	0.0965	0.0530	0.3126	0.7584
2019	0.2188	0.0151	0.0549	0.0960	0.0514	0.3126	0.7488
2020	0.2081	0.0130	0.0556	0.0959	0.0519	0.3126	0.7370
2021	0.2462	0.0151	0.0594	0.0970	0.0545	0.3126	0.7849
2022	0.2312	0.0143	0.0683	0.1464	0.0488	0.3126	0.8217
2023	0.2231	0.0138	0.0749	0.1455	0.0475	0.3126	0.8174

**Note:** Soil tillage intensity was calculated using a fixed coefficient per unit cultivated area; therefore, its value remains constant across years. The total intensity includes chemical fertilizer, pesticide, plastic film, irrigation, diesel, and soil tillage.

Fertilizer-related intensity increased during the early and middle stages of the study period and then fluctuated after 2017. It rose from 0.1711 t CO₂-eq·ha ⁻ ¹ in 2006 to 0.2462 t CO₂-eq·ha ⁻ ¹ in 2021, before declining to 0.2231 t CO₂-eq·ha ⁻ ¹ in 2023. This pattern suggests that fertilizer use per unit area intensified during the expansion stage but began to moderate in recent years. The decline after 2021 indicates a positive change in nutrient input management, although fertilizer remains a major source of land-based emissions.

Pesticide-related intensity peaked earlier and then declined. It increased from 0.0120 t CO₂-eq·ha ⁻ ¹ in 2006 to 0.0197 t CO₂-eq·ha ⁻ ¹ in 2013, followed by a decline to 0.0138 t CO₂-eq·ha ⁻ ¹ in 2023. This decline indicates that pesticide input intensity was reduced even as agricultural production continued to grow, suggesting an improvement in pest management efficiency. However, because pesticide emissions accounted for a relatively small share of the total carbon footprint, the contribution of pesticide reduction to total mitigation was limited.

Plastic film intensity increased continuously from 0.0321 t CO₂-eq·ha ⁻ ¹ in 2006 to 0.0749 t CO₂-eq·ha ⁻ ¹ in 2023. This was one of the fastest-growing intensity indicators. The continuous increase suggests that plastic film use became more intensive per unit cultivated area, reflecting the increasing reliance on mulching technology under water-limited conditions. This trend also indicates that plastic film management is not only a pollution-control issue but also a carbon mitigation issue.

Irrigation intensity remained relatively stable or slightly decreased before 2021, but increased sharply after 2022. It declined from 0.1115 t CO₂-eq·ha ⁻ ¹ in 2006 to 0.0970 t CO₂-eq·ha ⁻ ¹ in 2021, then rose to 0.1464 t CO₂-eq·ha ⁻ ¹ in 2022 and 0.1455 t CO₂-eq·ha ⁻ ¹ in 2023. This increase was closely related to the statistical breakpoint in effective irrigated area caused by the application of the Third National Land Survey-based classification system [17,18]. Therefore, the post-2022 irrigation intensity should be interpreted as reflecting both actual irrigation infrastructure improvement and statistical reclassification. Because irrigation emissions are strongly associated with electricity use for pumping, changes in the power grid mix, pumping depth, irrigation technology, and water-use efficiency may affect actual emission intensity. Future research should refine irrigation emission accounting using region-specific electricity emission factors, actual pumping energy data, water resources statistics, and remote-sensing-based irrigation monitoring [19,20].

Diesel intensity fluctuated within a relatively narrow range. It increased from 0.0418 t CO₂-eq·ha ⁻ ¹ in 2006 to 0.0545 t CO₂-eq·ha ⁻ ¹ in 2021, before declining to 0.0475 t CO₂-eq·ha ⁻ ¹ in 2023. This suggests that mechanization-related emission pressure was relatively stable compared with fertilizer, irrigation, and plastic film.

Overall, the intensity analysis shows that the main challenge for future emission reduction is not only the total amount of agricultural inputs, but also the increasing carbon pressure per unit cultivated area. Fertilizer intensity has shown signs of moderation, and pesticide intensity has declined, but plastic film and irrigation intensity have increased markedly. Therefore, the future low-carbon transition of crop production in the study area should focus on water-saving irrigation, low-carbon pumping systems, improved plastic film recycling, biodegradable materials, and precise nutrient management.

## 4. Discussion

### 4.1. Changes in agricultural inputs and planting structure

The evolution of agricultural inputs in the study area reflects the interaction between production expansion, resource constraints, and the gradual transition toward more regulated input management. In the early and middle stages of the study period, increases in fertilizer, pesticide, diesel, and plastic film use were closely associated with the expansion of crop production and the pursuit of higher yields. Similar patterns have been observed in other agricultural regions where yield improvement has historically relied on the intensive use of external inputs [26,27].

After the mid-2010s, fertilizer, pesticide, and diesel use showed stabilization or decline, while crop output and agricultural output value continued to increase. This suggests that the production system may have entered a stage in which input growth became more controlled. The decline in fertilizer and pesticide use after 2017 coincided with the promotion of green agricultural development, soil testing-based fertilization, organic fertilizer substitution, and pest control improvement in China [28]. However, because this study does not conduct causal policy evaluation, these policy and technological factors should be regarded as possible explanations rather than direct causal evidence.

The continuous increase in plastic film use is a distinctive feature of arid and semi-arid agricultural systems. In water-limited regions, plastic film mulching plays an important role in conserving soil moisture, improving soil temperature, and supporting crop establishment [29–31]. Therefore, the increase in plastic film use reflects not only input intensification but also the structural adaptation of regional crop production to water scarcity. However, the environmental impacts of plastic film, including residual film accumulation, microplastic pollution, and end-of-life treatment, were not fully captured in the current carbon accounting framework. This indicates the need to combine carbon mitigation with plastic pollution governance in future agricultural management.

The planting structure was dominated by grain crops during the entire study period. The expansion of grain crop area and yield contributed strongly to the increase in regional agricultural output. However, because grain crops occupied the majority of cultivated land, they also had a disproportionate influence on total carbon emissions through tillage, fertilization, mechanization, and irrigation demand. This indicates that low-carbon transformation of grain production will be central to reducing agricultural emissions in the study area while maintaining food security [32–34].

### 4.2. Carbon footprint dynamics and emission source contributions

The total carbon footprint of agricultural inputs increased substantially from 2006 to 2023, but carbon intensity indicators changed in different directions. The increase in total CF and CFA indicates that absolute emissions and land-based emission pressure continued to rise. In contrast, the decline in CFY after the early stage suggests that crop production became more efficient in terms of emissions per unit yield. The continuous decline in CFE indicates improved carbon-economic efficiency, but this indicator should be interpreted cautiously because it may be influenced by agricultural price changes, market structure, and crop value composition.

This differentiated pattern shows that agricultural low-carbon transition cannot be evaluated using a single indicator. If only total emissions are considered, the study area appears to face increasing carbon pressure. If output-based or value-based intensity is considered, agricultural production shows signs of improved efficiency. Therefore, the combination of CF, CFY, CFA, and CFE provides a more comprehensive understanding of the relationship between agricultural expansion and carbon emission intensity [35].

Cropland tillage and chemical fertilizer application were the dominant sources of carbon footprint during the study period. This result is consistent with previous findings that land management and fertilizer input are major contributors to agricultural carbon emissions [36–38]. The large contribution of tillage reflects the importance of cultivated area and soil disturbance in regional-scale carbon accounting. However, this study used a fixed tillage coefficient due to data limitations. In reality, tillage-related carbon emissions may vary with soil type, tillage depth, tillage frequency, crop rotation, and soil organic carbon status. Therefore, future research should incorporate field-scale soil carbon monitoring and tillage-specific parameters.

Fertilizer remained the second-largest emission source. Although fertilizer-related emissions declined after 2021, the contribution of fertilizer to total emissions remained substantial. Improving fertilizer use efficiency, optimizing fertilizer structure, and promoting precise nutrient management are therefore important mitigation measures [39,40]. Because this study used a static fertilizer emission coefficient, it could not distinguish between different fertilizer types or account for changes in fertilizer structure over time. Future studies should incorporate fertilizer-type-specific emission factors and direct N₂O emissions from fertilizer application.

Irrigation-related emissions increased sharply in 2022 and 2023 due to the reported expansion of effective irrigated area. However, this increase should be understood in the context of a statistical breakpoint. The official application of the Third National Land Survey results and the updated land-use classification system led to the re-identification and reclassification of irrigated farmland [17,18]. Therefore, the abrupt increase in effective irrigated area mainly reflects a statistical break adjustment rather than a sudden one-year physical expansion of irrigation or groundwater pumping. At the same time, high-standard farmland construction, water-saving irrigation projects, and irrigation district improvement contributed to the actual enhancement of irrigation capacity [21,22].

This distinction is important for interpreting irrigation-related carbon emissions. Since the irrigation coefficient used in this study is linked to effective irrigated area, the statistical adjustment directly increased calculated irrigation emissions in 2022 and 2023. External consistency checks, such as the moderate change in agricultural water use and the stable improvement of irrigation water-use efficiency, suggest that the increase in effective irrigated area was not accompanied by an equivalent surge in water consumption [19,20]. Nevertheless, the results still highlight the importance of the water–energy–carbon linkage in arid and semi-arid agriculture. Future research should improve irrigation emission accounting by incorporating regional electricity emission factors, actual irrigation energy consumption, pumping depth, and remote-sensing-based irrigation monitoring.

### 4.3. Emission reduction pathways for low-carbon crop production

Under the dual-carbon goals, agricultural emission reduction in the study area should consider both regional production characteristics and resource constraints [41]. Based on the results, the first priority is to reduce tillage-related carbon pressure. Conservation tillage, reduced tillage, straw return, and soil organic carbon management can help reduce soil disturbance and improve soil carbon storage. However, such measures should be adapted to local soil conditions, crop types, and water availability.

Second, fertilizer management remains a key mitigation pathway. The results show that fertilizer-related emissions are a major component of the total carbon footprint. Therefore, soil testing-based fertilization, precise nutrient management, organic fertilizer substitution, controlled-release fertilizers, and improved fertilizer application timing can help reduce unnecessary fertilizer input while maintaining crop yield. These measures are particularly important for grain-dominated production systems.

Third, irrigation-related mitigation has become increasingly important. The sharp increase in irrigation emissions after 2022 suggests that water-saving and energy-efficient irrigation should be prioritized. However, because the 2022 increase was strongly affected by the statistical breakpoint in effective irrigated area, future mitigation policies should be based on more refined data on actual irrigation energy consumption, water-use efficiency, and pumping systems. Drip irrigation, sprinkler irrigation, low-pressure irrigation systems, efficient pumping equipment, and improved irrigation scheduling can reduce both water consumption and energy demand. In addition, the use of renewable energy for irrigation pumping may help reduce electricity-related emissions in the future.

Fourth, plastic film management should be strengthened. Because plastic film use increased continuously during the study period, emission reduction should not rely simply on reducing film use, which may affect crop production under water-limited conditions. Instead, integrated management should be adopted, including biodegradable film, thicker recyclable film, improved film recovery systems, and possible “one film for two years” practices. However, such practices should be validated through local field trials to ensure that they do not reduce crop yield or increase other environmental risks.

In addition to technical mitigation measures, the modernization of carbon monitoring and agricultural input management is also necessary. Digital tools such as remote sensing, smart irrigation meters, agricultural input databases, and regional carbon accounting platforms can improve the accuracy of data collection and support more precise policy implementation. These tools can help track fertilizer use, plastic film recycling, irrigation water use, and machinery operation intensity in a more timely manner. They would also provide stronger data support for future uncertainty analysis and scenario simulation.

Looking toward 2030, the low-carbon transition of crop production in the study area will depend on the combined effects of fertilizer reduction, water-saving irrigation, plastic film recycling, mechanization efficiency improvement, and cropping structure optimization. Although this study does not conduct a quantitative scenario projection, the historical trends indicate that controlling irrigation-related emissions and plastic film emissions will become increasingly important. Future scenario studies could compare business-as-usual, input-reduction, water-saving, and integrated low-carbon pathways to provide more direct support for regional agricultural carbon mitigation policies.

### 4.4. Limitations and future research

Several limitations should be acknowledged. First, this study is based on regional-scale statistical data and therefore focuses on temporal changes rather than spatial heterogeneity. County- or prefecture-level spatial differences, GIS-based mapping, and spatial driving mechanisms were not analyzed. Accordingly, the conclusions mainly reflect regional temporal dynamics.

In addition, the effective irrigated area series contains a statistical breakpoint in 2022. This breakpoint was mainly caused by the official application of the Third National Land Survey results and the updated land-use classification system [17,18]. Therefore, the effective irrigated area before and after 2022 may not be fully comparable under the same statistical basis. Although the 2022 value was retained because it comes from official statistical sources, irrigation-related carbon emissions after 2022 should be interpreted cautiously. Future studies should cross-check effective irrigated area using land change survey data, water resources bulletins, irrigation district records, farmland construction records, and remote sensing products [18–22].

Second, the study adopts an input-based carbon footprint accounting framework rather than a complete process-based LCA. The emission sources included in the calculation were limited to chemical fertilizers, pesticides, plastic film, diesel fuel, irrigation, and soil tillage. Important field emissions such as N₂O from fertilizer application and CH₄ from rice paddies were not included because long-term data on fertilizer type, soil conditions, water management, and rice paddy management were unavailable. Similarly, emissions from harvesting, threshing, weed control, crop residue management, plastic film disposal, and agricultural machinery manufacturing were not separately quantified.

Third, fixed emission coefficients were used to ensure temporal comparability from 2006 to 2023. However, actual emission factors may vary with the regional electricity mix, irrigation energy source, groundwater pumping depth, soil type, tillage depth, fertilizer composition, and technological progress. In particular, irrigation-related emissions may be affected by changes in electricity generation structure and pumping energy efficiency. Future studies should incorporate dynamic regional coefficients and field-measured energy data to improve accounting accuracy.

Fourth, the carbon footprint per unit agricultural output value may be influenced by market price changes and crop value structure. Therefore, the decline in CFE should be interpreted as an improvement in carbon-economic efficiency rather than direct evidence of equivalent physical emission reduction. Future research could use constant-price agricultural output values and decomposition methods to distinguish price effects from real environmental efficiency improvements.

Finally, future research should expand from regional statistical accounting to field-scale and crop-specific analysis. Crop-specific carbon footprints, irrigated versus rainfed systems, dominant cropping models, uncertainty assessment, sensitivity analysis, and 2030 scenario simulations should be further explored. In addition, soil carbon sequestration should be incorporated to evaluate the net carbon balance of agricultural systems more comprehensively.

## 5. Conclusions

Under China’s dual-carbon goals, this study analyzed the temporal evolution of input-related carbon footprints from major crop production in the Inner Mongolia Autonomous Region from 2006 to 2023. Based on regional statistical data and an input-based carbon accounting framework, six major emission sources were considered: chemical fertilizers, pesticides, agricultural plastic film, diesel fuel, irrigation, and soil tillage. The main conclusions are as follows.

First, agricultural input use showed a clear transition from rapid expansion to partial stabilization. Fertilizer, pesticide, and diesel consumption increased during the early and middle stages of the study period and then stabilized or declined after the mid-2010s. In contrast, plastic film use continued to increase, reflecting the structural dependence of arid and semi-arid crop production on soil moisture conservation. The reported effective irrigated area increased sharply in 2022, but this increase should be interpreted primarily as a statistical breakpoint caused by the official application of the Third National Land Survey results and the updated land-use classification system, rather than as a sudden one-year physical expansion of irrigation.

Second, the total carbon footprint of agricultural inputs increased from 448.88 × 10⁴ t CO₂-eq in 2006 to 720.07 × 10⁴ t CO₂-eq in 2023, an increase of 60.4%. The carbon footprint per unit planted area also increased, suggesting that land-based emission pressure intensified. However, the carbon footprint per unit crop yield generally declined after the early stage, and the carbon footprint per unit agricultural output value decreased by 66.8% over the study period. These results indicate that agricultural production showed improved output-based and economic carbon efficiency, although absolute emissions continued to rise.

Third, soil tillage and chemical fertilizer application were the dominant emission sources, accounting for the largest shares of the total carbon footprint. Irrigation-related emissions increased sharply after 2022, while plastic film emissions showed a continuous upward trend. These findings suggest that future emission reduction should prioritize reduced or conservation tillage, precise fertilizer management, water-saving and energy-efficient irrigation, and improved plastic film recycling or substitution.

Finally, this study provides a regional-scale assessment of input-related carbon footprint dynamics in an arid and semi-arid agricultural region. However, it does not represent a complete agricultural greenhouse gas inventory or a full process-based LCA. Direct field emissions such as N₂O and CH₄, plastic film end-of-life emissions, soil carbon sequestration, crop-specific differences, and spatial heterogeneity were not fully quantified. Future research should combine field measurements, crop-specific input data, spatial analysis, uncertainty assessment, and scenario modeling to evaluate the net carbon balance and low-carbon transition pathways of dryland agriculture more comprehensively.

## Supporting information

S1 Data Minimal dataset used for carbon footprint calculation and figure generation. The file includes agricultural input data, crop area and yield data, carbon footprint by source, carbon footprint indicators, and emission intensity data from 2006 to 2023.
(XLSX)
